# The Effect of Influenza Virus on the Human Oropharyngeal Microbiome

**DOI:** 10.1093/cid/ciy821

**Published:** 2018-11-15

**Authors:** Elisa Ramos-Sevillano, William G Wade, Alex Mann, Anthony Gilbert, Robert Lambkin-Williams, Ben Killingley, Jonathan S Nguyen-Van-Tam, Christoph M Tang

**Affiliations:** 1 Sir William Dunn School of Pathology, University of Oxford, United Kingdom; 2 King’s College London, United Kingdom; 3 hVIVO Services Limited, Queen Mary BioEnterprises Innovation Centre, United Kingdom; 4 Department of Infection and Acute Medicine, University College London Hospital, United Kingdom; 5 Faculty of Medicine & Health Sciences, University of Nottingham, United Kingdom

**Keywords:** microbiome, influenza, upper respiratory tract

## Abstract

**Background:**

Secondary bacterial infections are an important cause of morbidity and mortality associated with influenza infections. As bacterial disease can be caused by a disturbance of the host microbiome, we examined the impact of influenza on the upper respiratory tract microbiome in a human challenge study.

**Methods:**

The dynamics and ecology of the throat microbiome were examined following an experimental influenza challenge of 52 previously-healthy adult volunteers with influenza A/Wisconsin/67/2005 (H3N2) by intranasal inoculation; 35 healthy control subjects were not subjected to the viral challenge. Serial oropharyngeal samples were taken over a 30-day period, and the V1-V3 region of the bacterial 16S ribosomal RNA sequences were amplified and sequenced to determine the composition of the microbiome. The carriage of pathogens was also detected.

**Results:**

Of the 52 challenged individuals, 43 developed proven influenza infections, 33 of whom became symptomatic. None of the controls developed influenza, although 22% reported symptoms. The diversity of bacterial communities remained remarkably stable following the acquisition of influenza, with no significant differences over time between individuals with influenza and those in the control group. Influenza infection was not associated with perturbation of the microbiome at the level of phylum or genus. There was no change in colonization rates with *Streptococcus pneumoniae* or *Neisseria meningitidis*.

**Conclusions:**

The throat microbiota is resilient to influenza infection, indicating the robustness of the upper-airway microbiome.

## Teaser

Secondary bacterial infections cause significant morbidity following influenza virus infection. We show that influenza A virus infection does not have a major impact on the human pharyngeal microbiome, indicating that microbial communities are robust in this site.

Secondary bacterial infection is a major cause of the mortality and morbidity associated with the influenza virus [1]. Bacterial pneumonia caused millions of deaths during influenza epidemics in the 20^th^ century [2]. For example, 94% of a group of patients who died in the 1918 influenza pandemic had evidence of secondary bacterial pneumonia [3], while 28% of fatalities in New York associated with the 2009 H1N1 influenza pandemic had bacterial co-infections, with most diagnosed post-mortem [4].

Several pathogens cause bacterial infection following influenza. Co-infections with *Haemophilus influenzae* were so common that it was initially thought to be the cause influenza [5]. *Streptococcus pneumoniae* is the commonest influenza-associated infection [6], while cavitatory *Staphylococcus aureus* pneumonia is a severe influenza complication; secondary pneumonia with methicillin resistant Staphylococcus aureus emerged in the 2009 pandemic [7]. There is also an association between influenza A and *Neisseria meningitidis* disease [8].

Multiple mechanisms could explain the development of bacterial disease following influenza. The virus is cytolytic and induces epithelial damage, impairment of surfactant production and muco-ciliary clearance [9, 10]. Exposure of the basement membrane and matrix offers binding sites for bacteria [11]; glycan receptors can be revealed by viral and bacterial neuraminidases [12, 13]. Additionally, the increased secretion of galectin 1 and galectin 3 can favor the adhesion of *S. pneumoniae* and *N. meningitidis* [14, 15]. Murine models indicate that host sialic acid, released by neuraminidases, can also act as a nutrient for pneumococci in the upper airway, promoting bacterial spread [16].

The human microbiome is a complex community that has co-evolved with its host. A healthy microbiota protects against invasion by pathogenic bacteria through colonization resistance, in which the microbiota restricts the growth or attachment of pathogens, either by competing for nutrients and ecological niches, or by direct antagonism [17].

Several factors influence the microbiome, including age, smoking, and antibiotic treatment [18–20]. Furthermore changes in the microbiota are associated with airway disease [21]. For example, healthy volunteers were reported to have a more diverse nasopharyngeal microbiome compared with patients suffering from pneumonia [22], while a highly diverse community is found in patients with asthma, cystic fibrosis, or Chronic Obstructive Pulmonary Disease [23, 24]. However, little is known about the impact of viral infection on the microbiota. No specific profiles of bacteria in the upper respiratory tract (URT) were associated with any of 7 different viral infections, although an apparent decrease in the carriage of *Haemophilus* and *Neisseria* was observed after rhinovirus [25, 26].

To further understand secondary bacterial infections, we investigated the impact of the influenza virus on the human nasopharyngeal microbiome. Using 454 pyrosequencing, we characterized the microbiota over a 30-day period in 52 healthy adults challenged with influenza A H3N2 and in 32 non-challenged individuals. This approach allowed us to define changes in the human microbiome following an influenza challenge. Furthermore, we examined whether influenza affected the carriage of pathogens in the upper airways.

## MATERIALS AND METHODS

### Study Design

The study took place in a quarantine facility with informed consent from volunteers, in accordance with the Declaration of Helsinki and with United Kingdom regulatory and ethical (Institutional Review Board) requirements.

Volunteers were screened before study entry. In brief, volunteers were healthy, with no acute or chronic medical condition; were between the ages of 18 and 45; were not living with anyone at risk of influenza complications; and had not had an influenza vaccine in the last 3 years. Blood samples from volunteers were collected before quarantine entry.

**Figure 1.**
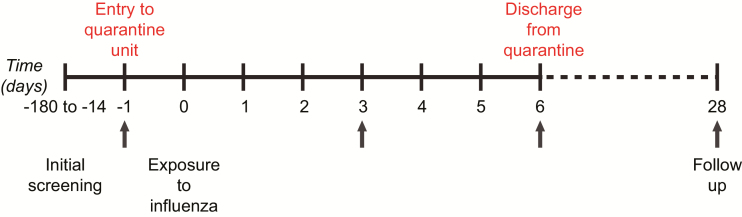
Study timeline. Volunteers were screened prior to admission to a quarantine unit on the day before receiving an intranasal challenge of influenza A (on day 0), then were followed for 28 days. The volunteers were kept in quarantine for 6 days post-inoculation. Controls were screened and housed in identical conditions, but were not subjected to a viral challenge. Throat swabs were collected at different time points during the study (ie, on days -1, 3, 6, and 28 post-inoculation).

Subjects were randomly allocated to either the challenge (n = 52) or control group (n = 35) on day -1. On day 0, individuals in the challenge group were inoculated intranasally with 5.5 log_10_ TCID50/ml/nostril of influenza strain A/H3N2/Wisconsin/67/2005. The control group members were not subjected to challenge (Figure 1).

### Clinical Monitoring and Sample Collection

Volunteers recorded symptoms daily, and vital signs were recorded 3 times daily. Nasopharyngeal swabs, oropharyngeal swabs, and venous blood were collected on days −1, 3, 6, and 28. Respiratory specimens were analyzed by quantitative polymerase chain reaction (qPCR) and serological specimens were analysed by hemagglutination inhibition (HAI) and microneutralization assays. Laboratory-confirmed influenza was defined as either a 4-fold or greater rise in HAI or microneutralization titres between day -2 and day 28 or a positive nasopharyngeal test by PCR.

Volunteers were classified according to symptoms and signs. Influenza-like illness (ILI) was defined, according to the Centers for Disease Control and Prevention definition, as an illness lasting ≥24 hours with either a temperature of >37.9°C or 2 or more symptoms, at least 1 of which was a respiratory symptom. Volunteers were labeled as symptomatic if they experienced symptoms with no fever; experienced a single symptom; had ≥2 symptoms, none of which were respiratory; or had an illness of <24 hours. Therefore, a volunteer could have an ILI or be symptomatic and not be infected with influenza. Individuals were classified according to their symptomatology and tests into 3 groups: the ILI^flu+^ group consisted of subjects who received the viral challenge and had an ILI, with evidence of an influenza infection (n = 19); the S^flu+^ group consisted of subjects who received the viral challenge and were symptomatic, with evidence of an influenza infection (n = 14); and the ILI/S^flu+^ group consisted of individuals belonging to both these groups (n = 33). Those who were not challenged with influenza and remained asymptomatic/influenza-negative formed the AC (asymptomatic control) group (n = 24).

### DNA Isolation and Pyrosequencing

DNA was extracted from swabs using the MoBio Power Soil isolation kit (MoBio laboratories). Barcoded primers were used to amplify ~500 bp from the V1-V3 region of the 16S ribosomal RNA (rRNA) gene, as previously described [27]. Amplification conditions were 5 minutes at 95°C, then 25 cycles of 95°C for 45 seconds, 53°C for 45 seconds, and 72°C for 1 minute 30 seconds, with a final step of 72°C for 15 minutes. The products were analyzed using an Agilent 2100 Bioanalyzer after purification with QIAquick (Qiagen) and were quantified by Quant-iT Picogreen (Invitrogen). The samples were pooled at equimolar concentrations and sequenced using a Roche 454 GS-FLX Titanium platform.

### Sequence Analysis

The quality of sequences was assessed using FastQC v0.11.3 (Babraham Institute). The pre-processing of sequences was performed with mothur v1.35.1 [28]. Next, we used trim.flows to remove sequences with mismatches in the primer sequence or barcode. Sequences were de-noised by shhh.flows, trimmed to remove primers and barcodes, then aligned to the SILVA 16S rRNA reference alignment using trim.seqs; chimeric sequences were eliminated by Uchime [29]. Sequences were used to interrogate the Human Oral Microbiome Database (HOMD) v13.2 at the 98.5% identity level using Basic Local Alignment Search Tool (BLAST). met the following criteria: read length >375 nucleotides (nt). (excluding barcode and primers), no ambiguous bases, or homopolymers of >8 nt.

A sequence dissimilarity distance of 0.015 was used to cluster sequences into operational taxonomic units (OTUs), using the average neighbor algorithm. Taxonomies were assigned using the HOMD reference dataset. The richness of communities was calculated using the Chao1 and Catchall indices [30, 31] and diversity was estimated with the Simpson’s inverse [32] and Shannon [33] indices. Good’s non-parametric estimator was used to assess the coverage of communities in samples [34]. The species diversity was examined by analyzing the beta diversity. Samples were adjusted to 2997 reads per sample by random sampling. Distance matrices were generated with the Jaccard Index and the thetaYC measure of dissimilarity [35], and by principal coordinate analysis (PCoA). Dendrograms, representing the relationships between samples, were analyzed by parsimony and UniFrac [36].

### *S. pneumoniae* and *N. meningitidis* Carriage

*N. meningitidis* was isolated by plating to GC selective medium (Oxoid). A 413 bp fragment-encoding RplF was amplified, sequenced, and identified with PubMLST [37]. Meningococcal isolates were characterized by serogrouping, serotyping, and serosubtyping. To detect the pneumococcus, *lytA* qPCR was performed, as previously described [38].

### Statistical Analyses

An analysis of alpha diversity was performed using the Kruskal-Wallis test with Dunn’s correction for multiple comparisons. An analysis of molecular variance was used to evaluate differences in PCoAs [39]. Parsimony tests were implemented by mothur [40]. The linear discriminant analysis (LDA) effect size was used to examine differences in OTUs in the control/challenged groups [41]; the alpha values were set to 0.05 and a LDA score of 2.5 was selected. A 2-tailed Fisher exact test was applied to compare colonization rates.

## RESULTS

### Infection and the Development of Symptoms After Influenza Challenge

Although volunteers were screened for antibodies against influenza in advance, 3 individuals in the challenge group and 3 in the control group had serological evidence of a prior H3N2 infection at study entry, so were excluded from subsequent analyses.

Influenza infection was confirmed in 43 of the 52 individuals (82%) subjected to the influenza challenge; 33 subjects developed symptoms, including 19 with ILI (ILI^flu+^). An additional 3 subjects were challenged with influenza and became symptomatic, but had no evidence of influenza infection. There were 3 other challenged individuals that remained asymptomatic, with no evidence of influenza.

### Characterization of the Pharyngeal Microbiome

A total of 2505196 sequences were obtained from 87 individuals (52 challenged and 35 controls) on days -1, 3, 6, and 28. DNA extraction failed for 1 sample and amplification was unsuccessful from 4 samples, giving us 343 usable samples. The removal of chimeric sequences yielded 2057548 sequences.

Initially, we compared ILI/S^flu+^ (n = 33) and AC (n = 24) individuals. To normalize the dataset, we took 2997 sequences from each sample; 11 samples had fewer than this, so were excluded, leaving 213 samples. We identified 2209 OTUs (based on <98.5% of identity, >4 representatives), with 24% of sequences falling into 3 OTUs: OTU1 (accounting for 9.78% of sequences), with predominant member *Fusobacterium periodonticum*; OTU2 (7.8%), with predominant members *S. salivarus*/*S. vestibularis*; and OTU3 (7%), with predominant members *S. mitis*/*S. pneumoniae*.

### Changes in the Diversity of Nasopharynx Microbiota Based on Operational Taxonomic Units Analysis

**Figure 2.**
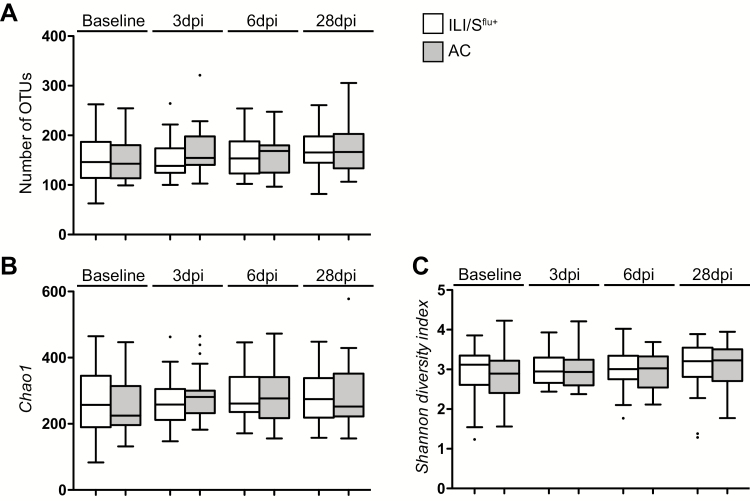
Analysis of the richness of the URT microbiome. The diversity of the microbiome was analyzed by sequencing the V1-3 region of 16S rRNA amplified from samples obtained from the ILI/S^flu+^ (white boxes) and asymptomatic control (grey boxes) groups following the influenza A challenge. The box whisker plots extend from the 25^th^ to 75^th^ percentiles, and the ends of the whiskers show the maximum and minimum values. The line in the middle of the box represents the median and the dots represent the outliers 1.5 greater or lower than the interquartile distance. The analysis was based on: (*A*) the number of OTUs; (*B*) Chao1 index; and (*C*) Shannon diversity index at baseline, 3 (3dpi), 6 (6dpi) and 28 (28dpi) days post–influenza challenge. Abbreviations: AC, asymptomatic control; dpi, days post–influenza challenge; ILI/S^flu+^, subjects who received viral challenge, had laboratory evidence of influenza, and had an influenza-like illness/were symptomatic; OTU, operational taxonomic units; URT, upper respiratory tract.

Coverage of bacterial communities was high (mean 97.6% ± 0.007%), with samples having an average of 161 OTUs (range 63 to 321). The number of OTUs at each time point was not significantly different between groups (asymptomatic controls vs ILI/S^flu+^, *P =* .4425; Figure 2). Furthermore, the number of OTUs remained remarkably constant over time, even in the ILI^flu+^ group, with no significant difference compared with asymptomatic controls (Supplementary Figures 1 and 2; *P*s = .5543 and .5836, respectively). The Chao 1 index measures the predicted richness of communities, so gives a higher value (median of 262 OTUs; range, 84 to 577 OTUs; Figure 2) than the recorded OTUs. The non-parametric estimator Catchall yielded a median of 437 OTUs per sample (range 66 to 2643). There were no significant differences in the richness of the between the ILI/S^flu+^ group and asymptomatic control groups (Figure 2). Furthermore, there was no significant difference in the richness of microbial communities in symptomatic individuals, even when comparing the ILI/S^flu+^ group with the AC group (Supplementary Figures 1 and 2).

We also estimated the diversity of communities using the Shannon index. Again, no difference was detected between the challenged and control groups (Figure 2; Supplementary Figure 1 and 2), even when we analyzed the diversity of the communities in highly-symptomatic ILI subjects and asymptomatic controls (Supplementary Table 1).

Next, we assessed differences between samples. Dendrograms were generated, highlighting similarities of the membership and structure of communities using Jaccard and thetaYC indices, respectively. At the 4 time points, results for certain individuals clustered together (eg, individual S^flu+^ individual 2, ILI^flu+^6, S^flu+^7, S^flu+^10, ILI^flu+^11, S^flu+^12, ILI^flu+^13, S^flu+^17, ILI^flu+^28, AC61, and AC65), indicating that the individual had a stronger influence on the microbiome than did influenza, with samples from 4 time points clustering together (Supplementary Figure 3). There was no significant clustering of OTUs either between groups or over time when we applied a parsimony test. The thetaYC index yielded a complex dendrogram (Supplementary Figure 4). Although there were no large shifts in the communities, minor changes were detected when we applied a parsimony test in the ILI/S^flu+^ and AC groups, with slight differences on days 3, 6, and 28 (*P* < .02, *P* < .024, and *P* < .001, respectively). However, these differences were not found after applying a Unifrac unweighted test.

**Figure 3.**
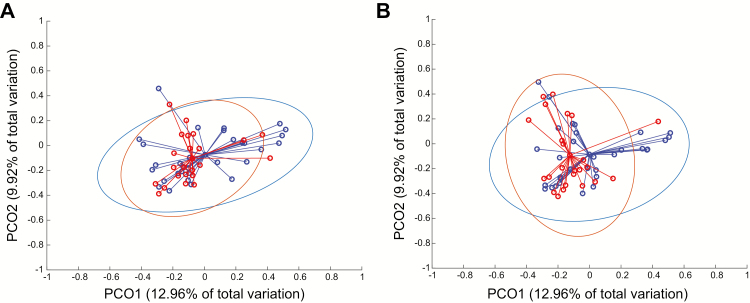
Throat microbiota structure in the ILI/S^flu+^ and asymptomatic control groups during the influenza challenge. The PCoA was based on the Thetayc index, comparing the community structures of samples from the ILI/S^flu+^ infected group (blue open circles) and asymptomatic control (red open circles) groups at days (*A*) 3 and (*B*) 6. The centroid represents the arithmetic mean for each of the groups, each dot represents the microbiota structure profile for each of the samples, while the ellipses represent the 95% of the samples belonging to each group. Abbreviations: ILI/S^flu+^, subjects who received viral challenge, had laboratory evidence of influenza, and had an influenza-like illness/were symptomatic; PCoA, principal co-ordinates analysis .

The community structure and membership was also visualized by PCoA (Figure 3). Small differences were found in the structure of communities in the ILI/S^flu+^ and AC groups at day 3 and day 6 (*P* < .035 and *P* < .033, respectively; Figure 3). Differences were not found in the memberships. The community structure was also examined for each group over time (Supplementary Figure 5); at day 6, the ILI/S^flu+^ group showed a statistically significant difference compared to baseline (*P* < .036), while in the AC group, days 3, 6, and 28 each showed some statistically significant difference compared to baseline (*P* < .001), indicating that quarantine might have effects on the URT microbiome.

**Figure 4.**
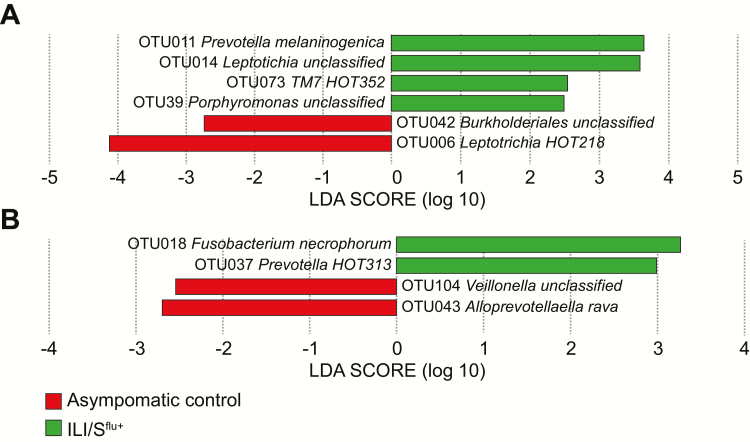
LEfSe analysis of abundant OTUs in ILI/S^**flu+**^ and asymptomatic controls. The positive scale indicates the LDA score (Log10) for the most abundant taxa in the ILI/S^flu**+**^ group (green bars), while the negative scale represents the LDA scores for the prevalent taxa in the asymptomatic control group on days (*A*) 3 and (*B*) 6. Abbreviations: ILI/S^flu+^, subjects who received viral challenge, had laboratory evidence of influenza, and had an influenza-like illness/were symptomatic; LDA, Linear Discriminant Analysis; LEfSe, LDA effect size; OTU, operational taxonomic units.

By LDA effect size analysis, the abundance of some OTUs was significantly different between groups on days 3 and 6 post-infection (Figure 4). The OTUs which were more abundant within the ILI/S^flu+^ group at day 3 included OTU11 (*Prevotella melaninogenica*), OTU14 (*Leptotrichia*), OTU38 (Human Oral Taxon 352), and OTU38 (*Porphyromonas*), while OTU42 (*Burkholdiales*) and OTU6 (*Leptotrichia* HOT218) were more abundant within the AC group. At day 6, OTU18 (*Fusobacterium necrophorum*) and OTU37 (*Prevotella*) were significantly more abundant in individuals in the ILI/S^flu+^ group.

### Alteration in the Ecology of the Pharyngeal Microbiome During Influenza

Next, we examined the communities by phylum. Of 2057548 sequences, 19416 could not be classified, with the remainder distributed into 11 phyla. A total of 97.1% of the sequences belonged to only 5 phyla: *Actinobacteria* (7.9%), *Fusobacteria* (24.2%), *Firmicutes* (36.5%), *Bacteroidetes* (14.1%) or *Proteobacteria* (14.4%). We detected *Spirochaetes*, *Synergistetes*, *Tenericutes*, GN02, and 2 uncultured prokaryotes (TM7 and SR1) each at <1% of the total microbiota.

**Figure 5.**
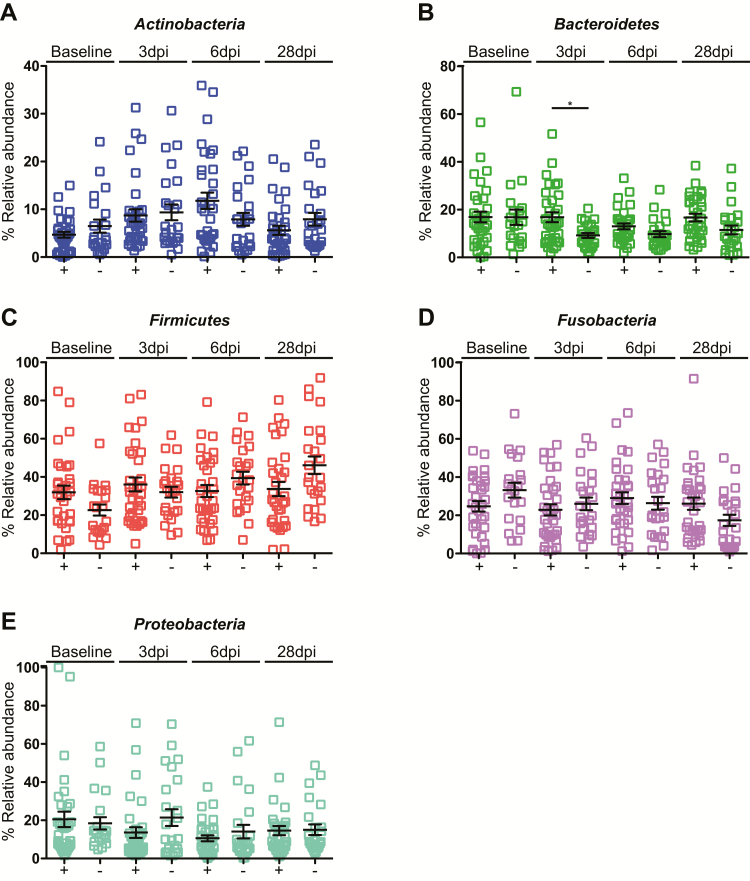
Relative abundance of common phyla in the human oropharynx. Comparison of the abundance of each phylum between individuals in the ILI/S^flu**+**^ group (+) and the asymptomatic controls (-). The phyla are indicated in each panel. Error bars represent the standard deviation. A non-parametric Kruskal-Wallis test for multiple comparisons was applied to identify the statistically significant differences in relative abundances between groups. *P < .05. Samples are from individuals prior to their entry to quarantine (baseline) and at days 3, 6, and 28 after the challenge, or not (in the control group). Abbreviations: dpi, days post–influenza challenge; ILI/S^flu+^, subjects who received viral challenge, had laboratory evidence of influenza, and had an influenza-like illness/were symptomatic.

**Figure 6.**
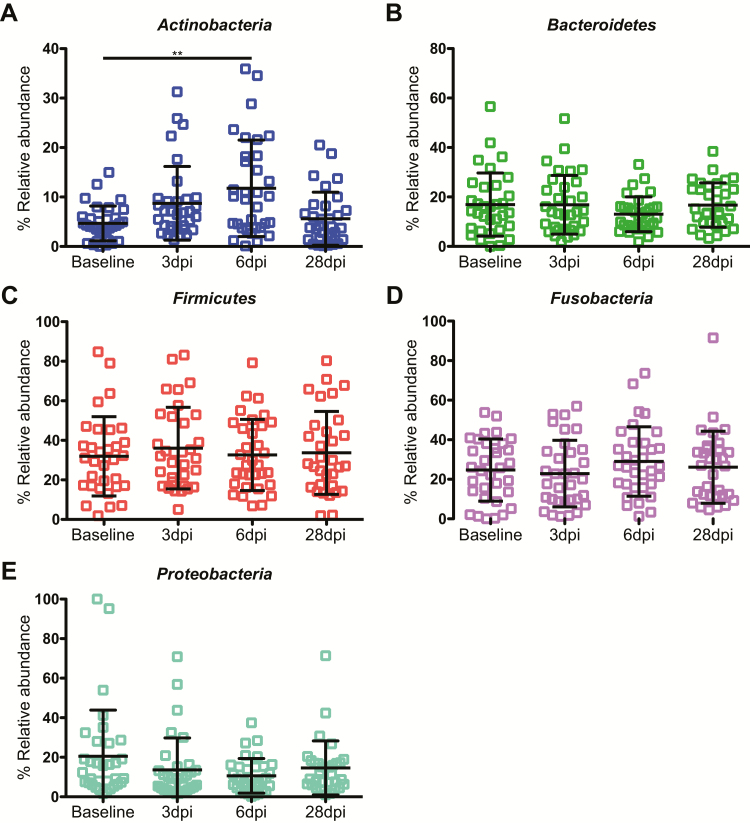
Relative abundance of common phyla in the human oropharynx. The abundance of phyla in individuals in the ILI/S^flu**+**^ group (+). Phyla are indicated in each panel and error bars represent the standard deviation. A non-parametric Kruskal-Wallis test for multiple comparisons was applied to identify the statistically significant differences in relative abundances between groups. *P < .05; **P < .01. Samples are from individuals prior to their entry to quarantine (baseline) and at days 3, 6, and 28 after the challenge. Abbreviations: dpi, days post–influenza challenge; ILI/S^flu+^, subjects who received viral challenge, had laboratory evidence of influenza, and had an influenza-like illness/were symptomatic.

The abundance of each phylum remained remarkably constant following the influenza challenge. There was a significant change in the *Bacteroidetes* levels, which increased in the ILI/S^flu+^ group at day 3 post-infection, as compared to the AC group (Figure 5). We also analyzed changes over the time. Within the ILI/S^flu+^ group, there was a significant change in the abundance of *Actinobacteria*, which increased by day 6 compared to baseline (*P* < .01) and returned to basal levels by day 28 (Figure 6). *Firmicutes* levels also increased between days 6 and 28 post-infection in the control group (Supplementary Figure 6), with an increase in *Streptococcus* (Supplementary Figure 7) that coincided with individuals returning to the community; this might reflect changes in social behaviors.

**Figure 7.**
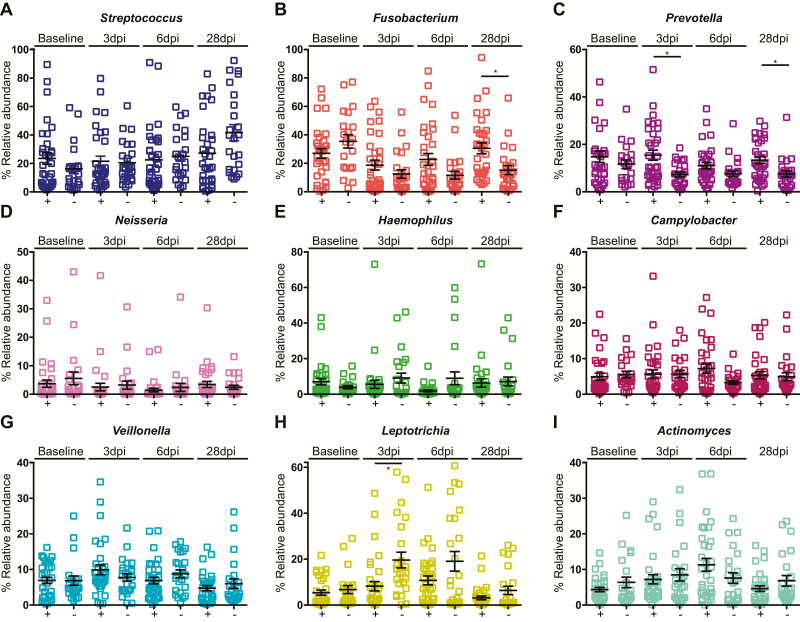
Relative abundance of the common genera in the human oropharynx. Comparison of the abundance of each phylum in individuals between the ILI/S^flu**+**^ group (+) and the asymptomatic controls (-). Genera are indicated in each panel. Error bars represent the standard deviation. A non-parametric Kruskal-Wallis test for multiple comparisons was applied to identify the statistically significant differences in relative abundances between groups. *P < .05. Samples are from individuals prior to their entry to quarantine (baseline) and at days 3, 6, and 28 after the challenge, or not (in the control group). Abbreviations: dpi, days post–influenza challenge; ILI/S^flu+^, subjects who received viral challenge, had laboratory evidence of influenza, and had an influenza-like illness/were symptomatic.

**Figure 8.**
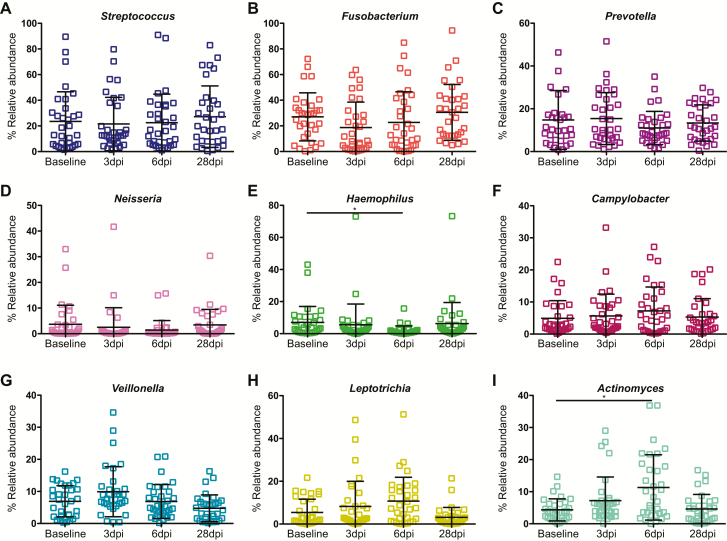
Relative abundance of the common genera in the human oropharynx. The abundance of each genera in individuals within the ILI/S^flu+^ group (+). The genera are indicated in each panel. Error bars represent the standard deviation. A non-parametric Kruskal-Wallis test for multiple comparisons was applied to identify the statistically significant differences in relative abundances between groups. *P < .05. Samples are from individuals prior to their entry to quarantine (baseline) and at days 3, 6, and 28 after the challenge. Abbreviations: dpi, days post–influenza challenge; ILI/S^flu+^, subjects who received viral challenge, had laboratory evidence of influenza, and had an influenza-like illness/were symptomatic.

With regard to the genus level, *Streptococcus* was a core component of the microbiome, accounting for 21.8% of bacteria, with *Fusobacterium* and *Prevotella* also abundant (comprising 15.4% and 9.9%, respectively). *Neisseria*, *Haemophilus*, and *Campylobacter* each accounted for 2–5% of the microbiota (Figure 7). Differences were identified between the ILI/S^flu+^ and AC groups (Figure 7), with a significant increase in the relative abundance of *Prevotella* on days 3 and 28 in the challenged group (*P* < .05) and an increase in *Fusobacterium* by day 28 in the challenged group (*P* < .05). Within the ILI/S^flu+^ group, a minor increase in *Actinomyces* was observed at day 6 post-infection (*P* < .05) and, in parallel, *Haemophilus* decreased (*P* < .01; Figure 8).

### *S. pneumoniae* and *N. meningitidis* Carriage Is Unaltered During Influenza

**Figure 9.**
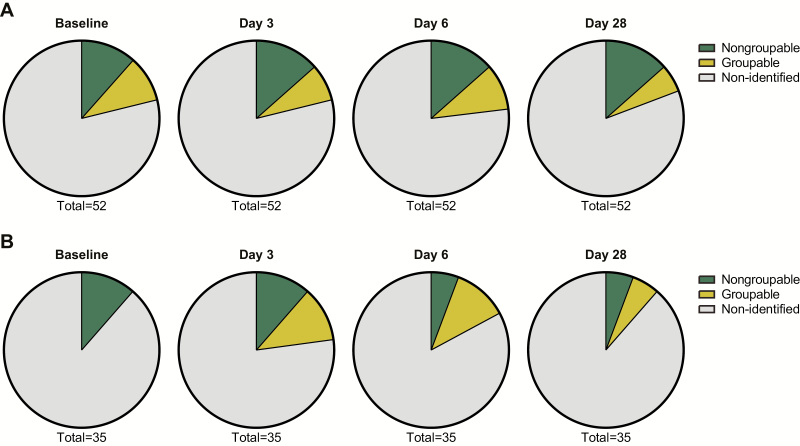
*Neisseria meningitidis* carriage following the influenza challenge. Meningococcal carriage in (*A*) the ILI/S^flu+^ group and (*B*) the asymptomatic control group at different times following the challenge. The carriage of non-groupable *N. meningitidis* was unchanged during influenza infection and ranged between 11.4 and 13.5%, except for control samples from days 6 and 28. There was no change in the carriage of groupable *N. meningitidis* over time or between groups. Abbreviation: ILI/S^flu+^, subjects who received viral challenge, had laboratory evidence of influenza, and had an influenza-like illness/were symptomatic.

As 16S sequencing often cannot discriminate different species, we analyzed the carriage of 2 important respiratory pathogens: *S. pneumoniae* and *N. meningitidis*. The rate of colonization by encapsulated or non-encapsulated *N. meningitidis* was similar, with no difference between challenged and control individuals (Figure 9). For *S. pneumoniae*, 16 of 36 influenza-challenged subjects (31%) and 12 of 23 controls (34%) were positive for *S. pneumoniae*, using qPCR, at day 3 (*P* = .8163).

## DISCUSSION

We performed the first prospective characterization of bacterial communities within the human URT during influenza infection. Previous studies have examined changes in the microbiota following challenges with other viruses [25, 26], and found only minor changes in certain bacterial genera. Furthermore, cross-sectional studies have characterized the microbiomes of patients with influenza [42]. However we are not aware of a previous study that examined individuals during a defined influenza challenge.

We detected slight perturbations in the upper respiratory microbiota following a challenge with influenza A. There was a high level of inter-individual variation in the upper respiratory microbiome, a common feature of human studies [27]. We found that the microbiota contains at least 11 phyla, with 5 (*Actinobacteria*, *Firmicutes*, *Fusobacteria*, *Bacterioidetes*, and *Proteobacteria*) constituting the core microbiome, as in a previous study [26], and with *Fusobacteria* highly prevalent (accounting for 24.2% of all OTUs) [20].

The microbiotas were dominated by *Streptococcus* and *Fusobacteria*, which include species that can cause influenza-associated pulmonary disease. However, during the acquisition of influenza, the profile of the URT microbiome remained remarkably stable. Although we detected no large shift in the bacterial population at the genus level, there was an increase in the abundance of *Prevotella* in the ILI/S^flu+^ group at days 3 and 28, a key time of secondary bacterial infection; although *P. melaninogenica* was associated with this increase, little is known about its role in respiratory disease.

Overall, our study does not provide evidence that influenza infection has a marked impact in shaping the pharyngeal microbiome. However, certain factors might be responsible for this. Participants were healthy adults, who do not generally suffer secondary bacterial infections; studies in at-risk individuals might reveal more significant changes in the microbiome, although they would pose serious ethical issues. In addition, other effects might become evident in a larger study. Furthermore, 16S rRNA–based methods are limited, lacking species-level resolution for particular genera, including *Streptococcus* and *Neisseria*. Even though cohort studies have established a relationship between influenza and the rate of pneumococcal carriage, we did not find differences in pneumococcal carriage following an influenza infection. For the meningococcus, we also examined capsule expression, as this virulence factor is only expressed by a subset of strains [43]. However, there was no alteration in the rate of carriage of encapsulated or non-encapsulated *N. meningitidis* with the advent of an influenza infection. We did not examine swabs for the presence of *S. aureus*, as this secondary bacteria pathogen follows influenza far less frequently than *S. pneumoniae* and is associated with severe disease rather than milder infections, such as that represented by our challenge.

In conclusion, our findings demonstrate that the upper airway microbiota is not reprogrammed by influenza infection, with only minor perturbations in bacterial communities, similar to recent findings with rhinovirus [44]. Further studies are required to examine the cross-kingdom interactions that are responsible for secondary bacterial infection being a key player in the mortality and morbidity associated with influenza.

## Supplementary Data

Supplementary materials are available at *Clinical Infectious Diseases* online. Consisting of data provided by the authors to benefit the reader, the posted materials are not copyedited and are the sole responsibility of the authors, so questions or comments should be addressed to the corresponding author.

ciy821_suppl_Supplementary-Table-1

## Notes

***Disclaimer.*** The views expressed in this manuscript are not necessarily those of the Department of Health, England.

***Financial support.*** This work was supported by an award from the Wellcome Trust (to C. M. T.’s laboratory). The quarantine challenge study was supported by a Cooperative Grant from US Centers for Disease Control and Prevention (grant number 1U01P000497-01).

***Potential conflicts of interest.*** All authors: No reported conflicts. All authors have submitted the ICMJE Form for Disclosure of Potential Conflicts of Interest. J. S. N.-V.-T. is currently on secondment to the Department of Health, England. Conflicts that the editors consider relevant to the content of the manuscript have been disclosed.
