# Supplementary material for: The Effect of Influenza Virus on the Human Oropharyngeal Microbiome
Source: Clin Infect Dis. 2018 Nov 15;68(12):1993–2002. doi: 10.1093/cid/ciy821 (PMC6541733; doi:10.1093/cid/ciy821)
Supplement: ciy821_suppl_Supplementary-Table-1 [file ciy821_suppl_supplementary-table-1.docx]

| Sample ID | Good’s coverage | No. of observed OTUs | Chao1 richness estimate | Simpson’s inverse diversity index | Shannon diversity index | Catchall richness estimate |
| --- | --- | --- | --- | --- | --- | --- |
| D10_28dpi | 0.979694 | 142.917 | 259.4444 | 15.150426 | 3.314312 | 501.2 |
| D10_3dpi | 0.979834 | 157.323 | 233.0389 | 13.601941 | 3.300068 | 343.5 |
| D10_6dpi | 0.984826 | 111.689 | 171.062 | 2.2624 | 1.768583 | 233.7 |
| D10_B | 0.973969 | 185.969 | 291.0885 | 9.783989 | 3.200211 | 432.4 |
| D11_28dpi | 0.973035 | 158.13 | 320.8115 | 7.933346 | 2.893876 | 1088.9 |
| D11_3dpi | 0.96922 | 190.272 | 346.2862 | 14.132138 | 3.398375 | 1141.4 |
| D11_6dpi | 0.967353 | 193.535 | 401.2071 | 14.693591 | 3.385971 | 931.8 |
| D11_B | 0.974321 | 176.114 | 301.7107 | 10.528489 | 3.327459 | 514.2 |
| D12_28dpi | 0.985247 | 165.611 | 192.3672 | 18.448833 | 3.623973 | 245.4 |
| D12_3dpi | 0.979748 | 124.701 | 230.449 | 9.089409 | 2.867624 | 358.4 |
| D12_6dpi | 0.970519 | 170.977 | 353.5311 | 10.869214 | 3.097337 | 2005.8 |
| D12_B | 0.991424 | 80.16 | 106.4564 | 5.37599 | 2.304128 | 125.1 |
| D13_28dpi | 0.976579 | 207.194 | 274.2912 | 7.515694 | 3.204471 | 328.6 |
| D13_3dpi | 0.973934 | 162.056 | 296.9163 | 10.629919 | 3.10393 | 425.2 |
| D13_6dpi | 0.975812 | 148.334 | 257.2268 | 10.662749 | 2.93752 | 346.1 |
| D13_B | 0.96942 | 218.649 | 343.307 | 9.667222 | 3.438822 | 546.4 |
| D14_6dpi | 0.965761 | 254.323 | 384.1355 | 21.769233 | 4.0229 | 619.9 |
| D14_B | 0.978307 | 146.209 | 254.7528 | 10.979693 | 3.155688 | 392.9 |
| D15_28dpi | 0.976026 | 166.624 | 287.7247 | 8.432461 | 3.113296 | 451.3 |
| D15_3dpi | 0.973588 | 175.515 | 297.0994 | 13.748873 | 3.44305 | 457 |
| D15_6dpi | 0.978043 | 163.583 | 263.6155 | 17.390549 | 3.562247 | 409.1 |
| D15_B | 0.970046 | 177.344 | 360.5718 | 8.327902 | 3.090279 | 606.1 |
| D17_28dpi | 0.983964 | 118.531 | 174.3706 | 7.445797 | 2.728756 | 245.7 |
| D17_3dpi | 0.983339 | 114.762 | 193.365 | 6.944487 | 2.661641 | 354 |
| D17_6dpi | 0.980587 | 127.534 | 233.7327 | 9.187328 | 2.898319 | 479.6 |
| D17_B | 0.985924 | 106.975 | 182.7224 | 6.261759 | 2.584922 | 347.7 |
| D19_28dpi | 0.981552 | 132.28 | 232.222 | 3.286295 | 2.277178 | 320.5 |
| D19_3dpi | 0.982877 | 132.484 | 200.774 | 5.521011 | 2.754658 | 276.1 |
| D19_6dpi | 0.977375 | 151.104 | 288.411 | 7.907278 | 3.026264 | 1008.7 |
| D19_B | 0.993378 | 62.908 | 83.57798 | 1.663629 | 1.234295 | 121.4 |
| D20_28dpi | 0.967377 | 180.408 | 388.7716 | 8.589633 | 3.037732 | 970.9 |
| D20_3dpi | 0.983409 | 116.072 | 194.1521 | 4.759365 | 2.503333 | 618 |
| D20_6dpi | 0.979887 | 121.445 | 246.2268 | 6.7794 | 2.615965 | 423.1 |
| D20_B | 0.984184 | 111.145 | 189.0644 | 2.485597 | 1.975178 | 325 |
| D22_28dpi | 0.974415 | 159.854 | 276.8294 | 5.725025 | 2.525784 | 481.9 |
| D22_3dpi | 0.973429 | 186.088 | 307.881 | 17.476084 | 3.549515 | 415.9 |
| D22_6dpi | 0.978326 | 139.432 | 241.5023 | 6.718887 | 2.545502 | 410.1 |
| D22_B | 0.983727 | 98.746 | 181.429 | 2.057703 | 1.542104 | 375.6 |
| D25_28dpi | 0.977115 | 192.232 | 279.0179 | 12.693301 | 3.576074 | 434.9 |
| D25_3dpi | 0.967667 | 188.866 | 371.8112 | 8.954267 | 3.099187 | 939.3 |
| D25_6dpi | 0.965419 | 219.293 | 388.9102 | 20.790432 | 3.74291 | 626.7 |
| D25_B | 0.977645 | 143.757 | 257.7891 | 6.292032 | 2.631322 | 763.2 |
| D26_28dpi | 0.985079 | 81.475 | 158.437 | 2.424133 | 1.381493 | 465 |
| D26_3dpi | 0.966433 | 221.636 | 354.6244 | 11.159926 | 3.427174 | 456.7 |
| D26_6dpi | 0.975156 | 166.377 | 284.3806 | 9.674869 | 3.143147 | 480.3 |
| D28_28dpi | 0.959249 | 241.441 | 444.7945 | 15.125283 | 3.58195 | 864.2 |
| D28_3dpi | 0.980561 | 135.13 | 221.3507 | 6.447943 | 2.661322 | 261.2 |
| D28_6dpi | 0.962029 | 204.716 | 448.2211 | 9.138437 | 2.973854 | 1214.2 |
| D28_B | 0.960211 | 221.304 | 465.1785 | 14.775715 | 3.48429 | 1189 |
| D29_3dpi | 0.98342 | 124.712 | 195.9958 | 5.122783 | 2.519773 | 273.3 |
| D29_6dpi | 0.974492 | 141.217 | 284.5673 | 3.698973 | 2.103463 | 789.4 |
| D29_B | 0.983673 | 117.586 | 189.46 | 9.468321 | 2.905281 | 331.5 |
| D2_28dpi | 0.979533 | 146.845 | 245.4594 | 6.728775 | 2.933907 | 406.3 |
| D2_3dpi | 0.980408 | 154.154 | 235.3445 | 4.396989 | 2.789865 | 281 |
| D2_6dpi | 0.967782 | 195.318 | 343.8904 | 7.291769 | 3.059883 | 579 |
| D2_B | 0.977339 | 168.767 | 268.6355 | 8.190837 | 3.206003 | 555.5 |
| D31_28dpi | 0.966761 | 200.828 | 387.05 | 11.407789 | 3.408548 | 689.5 |
| D31_3dpi | 0.979098 | 134.313 | 257.1091 | 7.061724 | 2.865651 | 425 |
| D31_6dpi | 0.970123 | 181.858 | 339.0116 | 11.793378 | 3.218203 | 723.9 |
| D31_B | 0.960186 | 213.293 | 437.6238 | 9.48195 | 3.118577 | 738.8 |
| D33_28dpi | 0.977047 | 154.803 | 284.6223 | 11.130366 | 3.278362 | 548.7 |
| D33_3dpi | 0.980548 | 132.315 | 210.6541 | 3.886251 | 2.438883 | 346.1 |
| D33_6dpi | 0.983437 | 118.934 | 175.0122 | 6.266426 | 2.483362 | 218.3 |
| D36_28dpi | 0.978202 | 189.617 | 265.0804 | 12.960032 | 3.501911 | 405.2 |
| D36_3dpi | 0.959529 | 264.195 | 463.1778 | 20.933378 | 3.930629 | 2484.6 |
| D36_6dpi | 0.968557 | 218.895 | 379.0209 | 16.841008 | 3.646934 | 2643.4 |
| D36_B | 0.974659 | 181.434 | 281.3731 | 9.22705 | 3.179238 | 363 |
| D37_28dpi | 0.964107 | 260.806 | 418.3748 | 10.58512 | 3.724709 | 864.4 |
| D37_3dpi | 0.971495 | 173.874 | 305.918 | 7.69444 | 2.94869 | 372.1 |
| D37_6dpi | 0.9813 | 121.325 | 206.4328 | 7.793531 | 2.743707 | 282 |
| D37_B | 0.976518 | 168.141 | 260.4286 | 12.470459 | 3.325646 | 344.1 |
| D39_28dpi | 0.976066 | 176.678 | 298.2414 | 20.533948 | 3.692877 | 566.6 |
| D39_3dpi | 0.972044 | 158.819 | 321.9347 | 8.561527 | 2.966396 | 540.5 |
| D39_6dpi | 0.971949 | 175.03 | 332.6555 | 11.654568 | 3.241436 | 626.4 |
| D3_3dpi | 0.986428 | 105.252 | 163.7723 | 6.402482 | 2.573888 | 408.6 |
| D3_6dpi | 0.980329 | 144.761 | 257.1192 | 15.212068 | 3.375176 | 848.9 |
| D3_B | 0.984024 | 132.028 | 210.9278 | 8.707367 | 3.01648 | 294.4 |
| D40_28dpi | 0.984376 | 95.744 | 160.4026 | 1.806535 | 1.283696 | 408.5 |
| D40_3dpi | 0.978913 | 148.439 | 241.8988 | 4.076064 | 2.563976 | 401.1 |
| D40_6dpi | 0.979504 | 154.312 | 246.2696 | 5.711603 | 2.867509 | 284.8 |
| D40_B | 0.987936 | 118.85 | 153.7039 | 8.083715 | 2.901832 | 185.2 |
| D41_28dpi | 0.982294 | 170.715 | 234.4106 | 25.683251 | 3.887855 | 324.5 |
| D41_3dpi | 0.982076 | 112.97 | 212.2246 | 7.033064 | 2.52601 | 640.5 |
| D41_6dpi | 0.979702 | 162.922 | 233.8495 | 14.117977 | 3.406012 | 314.3 |
| D41_B | 0.982002 | 114.936 | 216.8013 | 7.315999 | 2.691627 | 269.7 |
| D45_28dpi | 0.966665 | 213.784 | 372.3599 | 14.517388 | 3.515776 | 920.3 |
| D45_6dpi | 0.963874 | 206.73 | 428.1894 | 9.044669 | 3.202148 | 896.6 |
| D46_28dpi | 0.97529 | 194.587 | 290.6168 | 13.806615 | 3.521588 | 416.2 |
| D46_3dpi | 0.974785 | 147.823 | 277.8064 | 7.047102 | 2.747184 | 495.1 |
| D46_B | 0.960077 | 262.295 | 431.567 | 9.766315 | 3.57933 | 683 |
| D47_28dpi | 0.983317 | 124.036 | 191.3404 | 16.972958 | 3.27409 | 268.5 |
| D47_3dpi | 0.977288 | 137.699 | 286.1638 | 11.05586 | 3.008465 | 870.1 |
| D47_6dpi | 0.980075 | 136.584 | 247.4946 | 9.405806 | 2.989372 | 728.2 |
| D47_B | 0.984405 | 106.704 | 191.5132 | 3.827162 | 2.173619 | 387.8 |
| D48_28dpi | 0.977593 | 148.316 | 248.428 | 4.787549 | 2.514425 | 473.7 |
| D48_3dpi | 0.98462 | 100.226 | 191.407 | 4.91815 | 2.477405 | 417.4 |
| D48_6dpi | 0.981877 | 108.667 | 207.3023 | 8.441177 | 2.683581 | 331.2 |
| D48_B | 0.98422 | 143.814 | 196.7285 | 18.416251 | 3.521093 | 264.3 |
| D49_3dpi | 0.978114 | 134.851 | 295.2842 | 14.579597 | 3.19926 | 2279.1 |
| D49_6dpi | 0.985676 | 101.909 | 178.2922 | 10.44524 | 2.855721 | 282 |
| D49_B | 0.958252 | 261.417 | 464.9791 | 19.532485 | 3.85806 | 979.2 |
| D4_28dpi | 0.983279 | 155.299 | 203.25 | 7.984178 | 3.028871 | 261.8 |
| D4_3dpi | 0.974866 | 159.399 | 294.6902 | 13.595555 | 3.297436 | 677.8 |
| D4_6dpi | 0.985626 | 105.974 | 183.5095 | 8.715121 | 2.886467 | 302 |
| D4_B | 0.982696 | 113.412 | 205.6472 | 6.38493 | 2.585769 | 371.9 |
| D50_28dpi | 0.983697 | 120.898 | 187.4574 | 11.771253 | 3.09881 | 308.6 |
| D50_3dpi | 0.977014 | 137.131 | 260.1919 | 9.066427 | 2.956163 | 456.2 |
| D50_6dpi | 0.978212 | 152.177 | 250.9103 | 8.44941 | 3.162097 | 401.2 |
| D50_B | 0.967554 | 203.153 | 388.8667 | 12.294275 | 3.404944 | 2420.9 |
| D51_28dpi | 0.975358 | 165.425 | 271.2088 | 3.147865 | 2.387591 | 437.4 |
| D51_3dpi | 0.987981 | 105.766 | 146.817 | 8.386201 | 2.794314 | 177.3 |
| D51_6dpi | 0.977028 | 120 | 273.4126 | 5.569766 | 2.454241 | 699.4 |
| D51_B | 0.969558 | 179.548 | 341.3835 | 10.540487 | 3.15108 | 534.9 |
| D6_28dpi | 0.965565 | 217.099 | 357.5442 | 5.900344 | 2.975563 | 694.9 |
| D6_3dpi | 0.978855 | 138.671 | 249.5994 | 10.998851 | 3.045888 | 535.7 |
| D6_6dpi | 0.974128 | 163.529 | 298.5433 | 6.715666 | 2.781353 | 483.5 |
| D6_B | 0.972625 | 187.593 | 307.2203 | 12.89918 | 3.372375 | 509.6 |
| D7_28dpi | 0.961844 | 244.913 | 406.2516 | 20.626644 | 3.816933 | 597.4 |
| D7_3dpi | 0.967531 | 201.245 | 391.25 | 17.683465 | 3.602915 | 1111.7 |
| D7_6dpi | 0.979373 | 189.494 | 255.3573 | 19.164326 | 3.805832 | 362.2 |
| D7_B | 0.981585 | 126.854 | 226.6109 | 11.888271 | 3.098816 | 536.2 |
| R55_28dpi | 0.976961 | 170.051 | 283.7823 | 13.855343 | 3.432199 | 625.4 |
| R55_3dpi | 0.976719 | 149.189 | 292.008 | 10.769939 | 3.245319 | 540.2 |
| R55_6dpi | 0.970436 | 182.929 | 344.5992 | 23.286129 | 3.651831 | 582.5 |
| R55_B | 0.978769 | 196.915 | 262.3845 | 16.817529 | 3.858427 | 357.4 |
| R56_28dpi | 0.980155 | 131.951 | 225.8708 | 3.691686 | 2.348525 | 470.1 |
| R56_3dpi | 0.977856 | 148.149 | 236.9583 | 4.663473 | 2.453807 | 327.5 |
| R56_6dpi | 0.962646 | 231.198 | 418.6317 | 7.776194 | 3.31909 | 638.5 |
| R56_B | 0.98489 | 113.91 | 162.4623 | 2.613958 | 1.958878 | 219.7 |
| R59_28dpi | 0.97669 | 164.919 | 259.3008 | 6.228612 | 2.81401 | 371.9 |
| R59_3dpi | 0.964023 | 228.146 | 413.7545 | 13.417447 | 3.545959 | 527.6 |
| R59_6dpi | 0.975318 | 180.365 | 280.3969 | 12.641073 | 3.363034 | 472.5 |
| R59_B | 0.979681 | 149.007 | 225.4469 | 9.038825 | 3.075957 | 303.3 |
| R60_28dpi | 0.97958 | 183.39 | 235.3141 | 13.787392 | 3.475783 | 301.8 |
| R60_3dpi | 0.969705 | 206.254 | 320.7853 | 5.487088 | 2.872814 | 897 |
| R60_6dpi | 0.979402 | 169.57 | 239.5908 | 6.529806 | 2.997923 | 364.3 |
| R60_B | 0.975354 | 146.135 | 321.6861 | 5.18864 | 2.643196 | 925.5 |
| R61_28dpi | 0.961769 | 233.034 | 431.964 | 18.530668 | 3.684379 | 792.9 |
| R61_3dpi | 0.97541 | 174.054 | 273.3566 | 3.015968 | 2.4297 | 482 |
| R61_6dpi | 0.967232 | 205.124 | 392.0491 | 9.524361 | 3.300515 | 1765.3 |
| R61_B | 0.9755 | 194.179 | 273.3903 | 17.785233 | 3.601451 | 398.8 |
| R62_28dpi | 0.979707 | 173.976 | 234.4931 | 9.43758 | 3.20645 | 284.1 |
| R62_3dpi | 0.977031 | 140.885 | 254.1386 | 5.477696 | 2.596257 | 386.2 |
| R62_6dpi | 0.975536 | 154.164 | 257.4101 | 3.910067 | 2.389978 | 580.4 |
| R62_B | 0.961216 | 230.319 | 449.2299 | 19.186078 | 3.693859 | 753.3 |
| R63_28dpi | 0.986241 | 106.462 | 155.488 | 2.194729 | 1.802535 | 202.3 |
| R63_3dpi | 0.958292 | 321 | 464.5185 | 24.47064 | 4.211059 | 650.4 |
| R63_6dpi | 0.972836 | 176.116 | 319.994 | 14.543593 | 3.456661 | 750.9 |
| R63_B | 0.971727 | 173.701 | 322.337 | 6.573947 | 2.893972 | 498.5 |
| R64_28dpi | 0.980496 | 112.07 | 214.3658 | 2.756184 | 1.770911 | 731.6 |
| R64_3dpi | 0.973964 | 156.523 | 296.9836 | 7.976419 | 2.903784 | 567.9 |
| R64_6dpi | 0.97572 | 176.908 | 276.7378 | 11.070603 | 3.327373 | 389.5 |
| R64_B | 0.982934 | 102.199 | 195.6717 | 3.425473 | 2.082926 | 975.4 |
| R65_28dpi | 0.96853 | 225.026 | 371.9869 | 23.323364 | 3.946146 | 444.4 |
| R65_3dpi | 0.974443 | 154.644 | 298.294 | 9.622859 | 3.008362 | 650.5 |
| R65_6dpi | 0.976441 | 164.119 | 293.2271 | 13.059852 | 3.30568 | 309.5 |
| R65_B | 0.981933 | 113.209 | 206.8007 | 1.966296 | 1.560273 | 471.8 |
| R66_28dpi | 0.965143 | 230.969 | 398.9497 | 15.779893 | 3.677721 | 742.1 |
| R66_3dpi | 0.976358 | 160.093 | 281.8984 | 13.486922 | 3.239733 | 575 |
| R66_6dpi | 0.982357 | 124.59 | 216.2256 | 7.271536 | 2.746288 | 487.6 |
| R66_B | 0.980584 | 142.745 | 213.1482 | 4.365715 | 2.54646 | 324.6 |
| R67_28dpi | 0.967515 | 212.009 | 366.6188 | 21.497249 | 3.753376 | 640.3 |
| R67_3dpi | 0.965273 | 228.323 | 383.5892 | 17.849918 | 3.699032 | 740.9 |
| R67_6dpi | 0.95842 | 247.844 | 473.9372 | 13.618215 | 3.652935 | 1332.6 |
| R67_B | 0.981039 | 138.766 | 214.0404 | 12.837275 | 3.141908 | 267.5 |
| R70_28dpi | 0.948284 | 305.497 | 576.9502 | 17.165003 | 3.87402 | 1808.1 |
| R70_3dpi | 0.962422 | 221.372 | 436.7108 | 17.809677 | 3.596835 | 715 |
| R70_6dpi | 0.966831 | 178.419 | 415.7197 | 14.213953 | 3.261698 | 1449 |
| R70_B | 0.976706 | 148.784 | 273.7717 | 4.265721 | 2.536773 | 514.8 |
| R71_28dpi | 0.973061 | 203.338 | 299.6185 | 15.963839 | 3.517331 | 468.2 |
| R71_3dpi | 0.97814 | 120.074 | 261.6264 | 5.366489 | 2.425181 | 1225.3 |
| R71_6dpi | 0.985357 | 115.793 | 179.6968 | 5.922048 | 2.692961 | 226.8 |
| R71_B | 0.985844 | 103.834 | 184.9658 | 5.90116 | 2.484941 | 388.9 |
| R72_28dpi | 0.979891 | 151.286 | 242.8992 | 7.763189 | 3.051634 | 395.4 |
| R72_3dpi | 0.977012 | 197.835 | 292.0124 | 27.714833 | 3.975829 | 465.1 |
| R72_6dpi | 0.972335 | 194.205 | 341.8554 | 19.702749 | 3.687847 | 693.9 |
| R72_B | 0.983432 | 140.013 | 197.5051 | 6.62069 | 2.963822 | 244 |
| R74_28dpi | 0.978006 | 173.136 | 275.4028 | 9.056272 | 3.250382 | 486.8 |
| R74_3dpi | 0.979756 | 140.291 | 225.891 | 8.209866 | 2.857252 | 298.3 |
| R74_6dpi | 0.986188 | 96.561 | 156.2284 | 5.080221 | 2.417313 | 229 |
| R74_B | 0.986773 | 100.609 | 158.443 | 6.139507 | 2.452258 | 323.3 |
| R75_28dpi | 0.981615 | 132.823 | 242.3131 | 13.917829 | 3.258823 | 335.6 |
| R75_3dpi | 0.978727 | 140.879 | 229.1781 | 3.856859 | 2.377596 | 517.4 |
| R75_6dpi | 0.981687 | 118.954 | 209.3752 | 5.010603 | 2.358114 | 320.9 |
| R75_B | 0.974768 | 162.024 | 302.7988 | 14.374291 | 3.292727 | 559.1 |
| R76_28dpi | 0.982244 | 139.769 | 205.3849 | 11.267666 | 3.050388 | 234.2 |
| R76_3dpi | 0.9783 | 142.86 | 237.6447 | 9.674633 | 2.936872 | 672.5 |
| R76_6dpi | 0.966556 | 172.525 | 398.131 | 7.263271 | 2.723241 | 824.2 |
| R76_B | 0.964652 | 254.251 | 410.772 | 37.275739 | 4.226534 | 1660 |
| R78_28dpi | 0.980176 | 134.508 | 207.3676 | 7.107827 | 2.6175 | 313.1 |
| R78_3dpi | 0.985162 | 103.027 | 183.0455 | 6.155268 | 2.697453 | 254.2 |
| R78_6dpi | 0.978235 | 140.378 | 255.9347 | 8.229704 | 2.947545 | 384.3 |
| R79_28dpi | 0.967337 | 201.857 | 390.3039 | 13.037186 | 3.445163 | 857.4 |
| R79_3dpi | 0.975439 | 155.874 | 281.8718 | 11.516091 | 3.15256 | 504 |
| R79_6dpi | 0.980472 | 124.524 | 216.0896 | 5.386881 | 2.42008 | 369.6 |
| R80_28dpi | 0.981472 | 124.971 | 183.1765 | 3.691684 | 1.989051 | 232.9 |
| R80_3dpi | 0.981762 | 147.333 | 215.587 | 7.854118 | 3.069755 | 254.5 |
| R80_6dpi | 0.984413 | 130.063 | 184.0068 | 9.711219 | 3.058014 | 219.8 |
| R81_28dpi | 0.975196 | 168.134 | 273.726 | 8.653246 | 3.040824 | 372.8 |
| R81_3dpi | 0.976011 | 160.403 | 290.0474 | 10.082753 | 3.068222 | 854.3 |
| R81_6dpi | 0.975483 | 170.956 | 262.8262 | 6.484133 | 2.884156 | 427.5 |
| R81_B | 0.978411 | 142.689 | 253.57 | 11.589039 | 3.080636 | 764.2 |
| R82_28dpi | 0.974015 | 158.302 | 299.1628 | 6.24838 | 2.670771 | 632.7 |
| R82_3dpi | 0.979399 | 122.916 | 230.8818 | 7.47788 | 2.815733 | 366.4 |
| R82_6dpi | 0.981613 | 114.259 | 213.1191 | 3.747603 | 2.116265 | 309.9 |
| R82_B | 0.982141 | 137.699 | 209.3701 | 3.441402 | 2.361665 | 314.6 |
| R85_28dpi | 0.982184 | 139.959 | 228.1619 | 12.146864 | 3.25466 | 391.4 |
| R85_6dpi | 0.978387 | 113.448 | 295.536 | 5.707053 | 2.492685 | 1929.6 |
| R85_B | 0.988358 | 98.985 | 131.1488 | 3.370622 | 2.0763 | 158.9 |
| R86_28dpi | 0.982311 | 128.865 | 220.6878 | 9.478479 | 3.007436 | 402.7 |
| R86_3dpi | 0.981729 | 135.185 | 192.2123 | 5.40566 | 2.583989 | 237.9 |
| R86_6dpi | 0.974434 | 167.279 | 276.5111 | 11.567422 | 3.207538 | 399.2 |
| R86_B | 0.970628 | 186.247 | 335.5217 | 8.951355 | 3.097365 | 751.8 |
